# Relating Antimicrobial Resistance and Virulence in Surface-Water *E. coli*

**DOI:** 10.3390/microorganisms11112647

**Published:** 2023-10-28

**Authors:** Connor D. LaMontagne, Elizabeth C. Christenson, Anna T. Rogers, Megan E. Jacob, Jill R. Stewart

**Affiliations:** 1 Department of Environmental Science and Engineering, Gillings School of Global Public Health, University of North Carolina, Chapel Hill, NC 27516, USA; cdl74@live.unc.edu (C.D.L.); elizabeth.christenson@duke.edu (E.C.C.); 2 Nicholas Institute for Energy, Environment, and Sustainability, Duke University, Durham, NC 27705, USA; 3 Kenan Institute for Ethics, Duke University, Durham, NC 27705, USA; 4 Department of Population Health and Pathobiology, College of Veterinary Medicine, North Carolina State University, Raleigh, NC 27607, USA; anna_rogers@med.unc.edu (A.T.R.); megan_jacob@ncsu.edu (M.E.J.); 5 Office of Genomics Research, Lineberger Comprehensive Cancer Center, University of North Carolina, Chapel Hill, NC 27516, USA

**Keywords:** virulence, antimicrobial resistance (AMR), surface water, commercial animal agriculture, *E. coli*

## Abstract

The role of the environment in the emergence and spread of antimicrobial resistance (AMR) is being increasingly recognized, raising questions about the public health risks associated with environmental AMR. Yet, little is known about pathogenicity among resistant bacteria in environmental systems. Existing studies on the association between AMR and virulence are contradictory, as fitness costs and genetic co-occurrence can be opposing influences. Using *Escherichia coli* isolated from surface waters in eastern North Carolina, we compared virulence gene prevalence between isolates resistant and susceptible to antibiotics. We also compared the prevalence of isolates from sub-watersheds with or without commercial hog operations (CHOs). Isolates that had previously been evaluated for phenotypic AMR were paired by matching isolates resistant to any tested antibiotic with fully susceptible isolates from the same sample date and site, forming 87 pairs. These 174 isolates were evaluated by conventional PCR for seven virulence genes (*bfp*, *fim*H, *cnf*-1, STa (*est*A), EAST-1 (*ast*A), *eae*, and *hly*A). One gene, *fim*H, was found in 93.1% of isolates. Excluding *fim*H, at least one virulence gene was detected in 24.7% of isolates. Significant negative associations were found between resistance to at least one antibiotic and presence of at least one virulence gene, tetracycline resistance and presence of a virulence gene, resistance and STa presence, and tetracycline resistance and STa presence. No significant associations were found between CHO presence and virulence, though some sub-significant associations merit further study. This work builds our understanding of factors controlling AMR dissemination through the environment and potential health risks.

## 1. Introduction

Antimicrobial resistance (AMR) is a leading threat to global health. If left unaddressed, this ability of microbial pathogens to survive treatment with previously effective antimicrobials may take more lives than cancer by 2050 [1]. A large body of work has been dedicated to understanding how AMR spreads in order to produce effective interventions. One key finding has been the importance of environmental pathways to this dissemination [2,3]. Hotspots of antimicrobial use—like hospitals, retirement homes, commercial animal farms, and tourist destinations—may pass substantial concentrations of antimicrobials and resistant microorganisms into waterways via wastewater treatment plants or runoff [4,5,6]. These organisms and antimicrobials, while potentially hazardous in their own right, may also promote AMR development in indigenous microorganisms in these waters through selection of naturally resistant organisms or horizontal gene transfer (HGT) [2].

While elevated environmental AMR is expected to increase the risk of resistant infections arising in humans, the extent of this relationship is unclear—a major evidence gap impeding a move toward best practices and regulation [7]. One unanswered question in understanding the risk posed by environmental resistance is the relationship between AMR and virulence, or the ability of a pathogen to infect and thrive in its host. Both AMR and virulence are necessary for a microorganism to be a resistant pathogen, so determining the relationship between the two is valuable for understanding how resistant pathogens emerge. Indeed, experts have called for research into the AMR–virulence link as an important contributor to human health risk assessments of environmental AMR [8].

Most work on the topic has focused on bacteria isolated directly from humans and animals, and even then, results have been inconsistent. A human population-based study found resistance to eight of twelve examined antibiotics to be more common in *Escherichia coli* containing virulence factors than those without, even when controlling for antibiotic use and symptomatic status in patients [9]. Work on uropathogenic *E. coli* found AMR to be significantly more common in pathogenic than non-pathogenic isolates [10]. For animals, research on swine with diarrhea in Ontario found enterotoxigenic *E. coli* (ETEC; i.e., virulent) to more commonly carry a gene for chloramphenicol resistance than non-ETEC, an effect that was not seen for kanamycin [11]. Such associations can theoretically be supported by the co-occurrence of specific virulence genes and antimicrobial resistance genes (ARGs) on mobile genetic elements like plasmids and integrons, as Travis et al. demonstrated for chloramphenicol and not kanamycin [11]. These co-occurring genes may be co-transferred between microorganisms via HGT. It is worth noting that clinical isolates are often taken from humans or animals exhibiting diarrhea (indicating virulence) without considering if these individuals were given antimicrobial treatment as a response to the symptom. Thus, without controlling for this confounder, positive associations between virulence and AMR in these cases should be interpreted with care.

Negative associations between AMR and virulence (i.e., resistant organisms being less likely to be virulent) have also been documented [12,13]. The most common justification for these associations is based on fitness cost. Plasmids are often metabolically costly to maintain, so if a cell needs multiple plasmids to exhibit both AMR and virulence, it may favor losing plasmids to improve chances of survival [14]. The value of AMR or virulence may outweigh these costs when encountering lethal concentrations of antimicrobials or infecting a host, both of which often occur in clinical scenarios. In the environment, however, these conditions are less common, so fitness costs may play a larger role there in determining the AMR–virulence relationship.

A far smaller body of research has begun to address the virulence–AMR relationship in the environment. Much of this work forgoes direct assessment of ARGs in favor of integrons—mobile genetic elements known for their close ties to AMR—finding integrons to correlate positively with select virulence genes [15,16]. Research on the river Rhine assessed AMR and virulence more directly, but was unable to establish any relationship, likely due to low measured prevalence of virulence genes [17]. A study of AMR and virulence in *E. coli* isolated from oysters and estuarine waters in Thailand found negative associations between sulfamethoxazole resistance and the toxin genes *lt* and *stx* [18]. Research on *E. coli* in spring water in the Eastern Indian Himalayas found a positive association between tetracycline resistance and the *elt* toxin gene and between quinolone resistance and the *eae* adhesin gene, along with a negative association between streptomycin resistance and two toxin genes [19]. Work on the Minjiang River found high levels of AMR, integron presence, and virulence, though the researchers did not assess associations between these outcomes [20]. Generally, these studies have not sought to directly correlate AMR and virulence, making it difficult to compare findings to those from clinical research.

To better understand the AMR–virulence relationship in the environment, we used *E. coli* isolated from surface waters in eastern North Carolina to compare virulence gene prevalence between isolates resistant and susceptible to antibiotics. Because the study area hosts some of the nation’s highest densities of commercial hog operations (CHOs), we also compared isolates from sites with or without upstream CHOs. We hypothesized that there would be a significant difference in virulence prevalence between resistant and susceptible isolates as well as between isolates from sites with or without upstream CHOs.

## 2. Materials and Methods

*Sampling, isolation, and AMR testing.* Water sampling, *E. coli* isolation and identification, and testing for antimicrobial resistance had been performed previously [21]. Briefly, sites were selected as per a previous United States Geological Survey study, based on whether they were downstream from a CHO (swine site) or not (background site) [22]. Water samples from these sites were filtered and the filters were used to inoculate selective mTEC media. After a multi-step incubation (37 °C for 2 h followed by 44 °C for 22 h (±2 h)), presumptive *E. coli* were picked from plates, streaked to isolation, and confirmed via the indole test. These culture steps suggested that all bacteria tested in the study were viable. Isolates were stored in tryptic soy broth with 20% glycerol solution at −80 °C. Antimicrobial resistance testing was performed using Kirby–Bauer disk diffusion method and interpreted as per 2017 CLSI human guidelines for the following 11 antibiotics: amoxicillin-clavulanate, ampicillin, cefoxitin, ceftriaxone, chloramphenicol, ciprofloxacin, gentamicin, imipenem, levofloxacin, tetracycline, and trimethoprim-sulfamethoxazole [23]. Instances of presumptive extended-spectrum beta-lactamase were confirmed both phenotypically and genotypically, and instances of multi-drug resistance, defined as resistance to three or more classes of antibiotics, were also noted [21]. Examples of Kirby–Bauer plates are provided in Supplemental Materials (Figure S1).

*Isolate pairing and DNA extraction.* To account for spatial and temporal variation, a subset of matched pairs was created (*n* = 254, 127 pairs) by matching each isolate resistant to at least one antibiotic to a completely susceptible isolate from the same sample date and site (i.e., same sample). If matching by site was not possible (48 of 127 pairs), isolates were picked from the same date in the closest watershed.

Isolates were streaked onto trypticase soy agar (TSA) and incubated for 24 h at 44 °C. A crude DNA extraction was then performed for each isolate by first boiling a loopful of cells in 100 µL sterile, nuclease-free water for 20 min. The samples were left at room temperature for 5 min, then centrifuged at 10,000 rpm for 30 s. Supernatants were collected and pellets were discarded. Samples were stored at −20 °C for up to 2 months, then moved to 4 °C for the duration of the analyses (~4 months).

*Virulence testing.* Conventional, single PCR was performed to determine the presence or absence of each of the following virulence genes, selected to include genes that were from various *E. coli* serotypes, important to both human and swine infections, and present on plasmids and/or chromosomes: *bfp*, *fim*H, *cnf*-1, STa (*est*A), EAST-1 (*ast*A), *hly*A, and *eae*. Each reaction had a 25 µL total volume consisting of 1 µL crude bacterial DNA extract, 0.4 µM primers, 9.5 µL sterile, nuclease-free water, and 12.5 µL GoTaq Colorless Master Mix (Promega, Madison, WI, USA). Reactions were carried out in a CFX96 thermocycler (Biorad, Hercules, CA, USA). Details of PCR running conditions can be found in Table 1 and primer details can be found in Table 2. Reaction products were confirmed via gel electrophoresis on a QIAxcel capillary electrophoresis machine (Qiagen, Hilden, Germany) against known gene weights.

*Dataset management and statistical analyses.* Multiple isolates from the same sample possessing identical AMR and virulence (as determined by the current work) profiles were assumed to be genetic clones. Groups of assumed clones were “collapsed” by choosing one representative via random number generator and removing the others, along with their respective matches (*n* = 174, 87 pairs; 67 swine pairs, 20 background pairs). Analyses of the non-collapsed dataset are included in the supplemental materials (Table S1).

Multiple logistic regressions were run in RStudio (version 3.4.3) using presence of virulence outcomes individually and in aggregate as well as presence of AMR and upstream presence of CHO as predictors. A test for multicollinearity among predictors was performed using Pearson’s r, with r > 0.5 considered moderately correlated and r > 0.8 considered highly correlated (Table S2).

Models were grouped to assess the association between (1) any virulence and any phenotypic resistance, (2) any virulence and each type of phenotypic resistance, (3) each type of virulence and any phenotypic resistance, and (4) each type of virulence and each type of phenotypic resistance (see Table S3 for key). Any virulence was defined as the presence of at least one virulence gene. Any phenotypic resistance was defined as the presence of at least one resistance phenotype, with resistance phenotype data being grouped by antibiotic class, which grouped cefoxitin with ceftriaxone (cephalosporins) and ciprofloxacin with levofloxacin (quinolones). In classes with multiple phenotypes tested, the presence of either of the phenotypes was considered positive. Because no isolates were resistant to imipenem or gentamicin in this dataset, those variables were removed from regressions that assessed each type of phenotypic resistance. Furthermore, predictors that exhibited complete or quasi-complete separation (i.e., the prevalence of these variables was too low for the regression to properly assess associations) were removed. In all cases, beta coefficients were exponentiated to produce odds ratios, and 95% confidence intervals were calculated via likelihood ratio (Supplementary Table S4).

## 3. Results

The results of sampling and AMR testing can be found in Christenson et al. (2022) [21]. In the original dataset, isolates with at least one resistance phenotype were detected more often in sites downstream of CHOs (19%; *n* = 556) than in background sites (6%; *n* = 356). Tetracycline resistance was the most common phenotype (17% CHO, 5% background), followed by ampicillin resistance (5% CHO, 0.8% background). Virulence results are presented here in Table 3. The most commonly found gene was *fim*H, in 93.1% of isolates. Excluding *fim*H, at least one virulence gene was found in 24.7% of isolates. Due to the high *fim*H prevalence, analyses were conducted both with and without *fim*H to avoid masking the results of the less common genes. When excluding *fim*H, a virulence gene was found in 17.2% of isolates resistant to at least one antimicrobial and in 32.2% of pan-susceptible isolates. Lower virulence prevalence among resistant compared to susceptible isolates was comparable among isolates from sites downstream of a CHO (18.6% and 31.3%) and background sites (11.8% and 34.8%), respectively.

**Figure 1:**
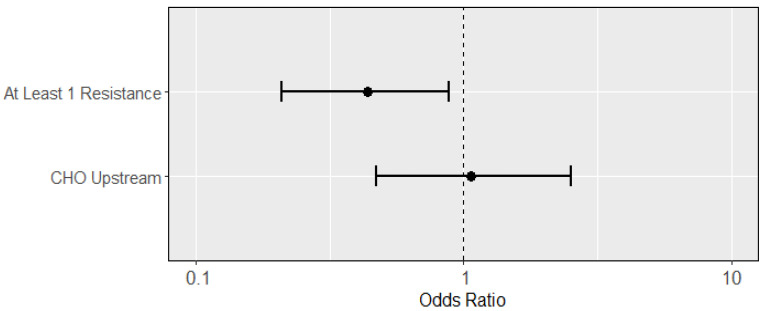
Odds ratios (dots; with 95% confidence intervals as lines) of predictors from multiple logistic regression for an isolate having at least one virulence gene. Presence of at least one resistance phenotype negatively predicted presence of at least one virulence gene (OR: 0.44; 95% CI: 0.21, 0.89) while presence of upstream CHO did not appear to predict (OR: 1.1; 95% CI: 0.47, 2.5) virulence presence.

The presence of any phenotypic resistance appeared to decrease the likelihood of possessing a virulence gene when excluding *fim*H (OR: 0.44; 95% CI: 0.21, 0.89; Figure 1). When including *fim*H, the effect remained negative but fell below statistical significance (OR: 0.58; 95% CI: 0.15, 2.01; Table S5). CHO presence, however, did not predict virulence (OR: 1.06; 95% CI: 0.47, 2.53), and this effect became negatively predictive when including *fim*H, though still statistically insignificant (OR: 0.33; 95% CI: 0.02, 1.84). Figure 1 displays the odds ratios of presence of resistance and presence of CHO predicting at least one virulence gene in the isolates.

**Figure 2:**
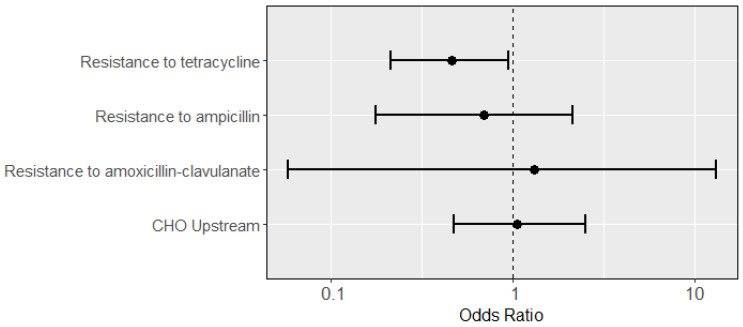
Odds ratios (dots; with 95% confidence intervals as lines) of predictors from a multiple logistic regression for an isolate having at least one virulence gene, using each type of resistance as a separate predictor (predictors exhibiting complete or quasi-complete separation were dropped). Presence of tetracycline resistance negatively predicted presence of at least one virulence gene (OR: 0.46; 95% CI: 0.22, 0.95), while virulence was not predicted by ampicillin resistance (OR: 0.70; 95% CI: 0.18, 2.16), amoxicillin-clavulanate resistance (OR: 1.31; 95% CI: 0.06, 13.2), or CHO presence (OR: 1.06; 95% CI: 0.48, 2.53).

When predicting the carriage of any virulence gene except *fim*H and including individual resistance phenotypes, we found that tetracycline resistance decreased the likelihood of virulence gene presence (OR: 0.46; 95% CI: 0.21, 0.95; Figure 2). When including *fim*H, this effect was no longer seen (OR: 1.52; 95% CI: 0.43, 6.17; Table S5). In both cases, ampicillin resistance (OR: 0.70; 95% CI: 0.18, 2.16) and amoxicillin-clavulanate resistance (OR: 1.31; 95% CI: 0.059, 13.2) were insignificant. Resistance to cefoxitin or ceftriaxone, chloramphenicol, ciprofloxacin or levofloxacin, sulfamethoxazole-trimethoprim, multi-drug resistance, and production of extended-spectrum beta lactamases (ESBL) were all dropped from the regressions, as they exhibited complete or quasi-complete separation. No substantial collinearity was found between the remaining predictors (Table S2). Again, CHO presence did not predict virulence (OR: 1.06; 95% CI: 0.48, 2.53).

**Figure 3:**
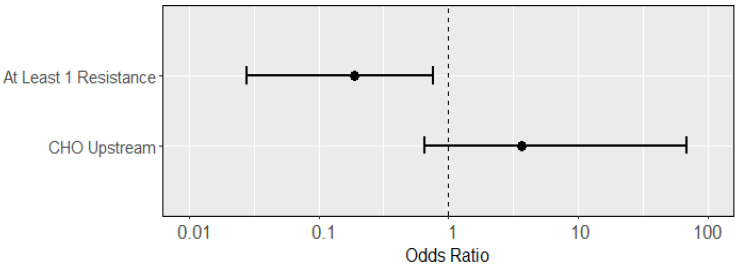
Odds ratios (dots; with 95% confidence intervals as lines) of predictors from a multiple logistic regression for an isolate having the STa virulence gene. Presence of at least one resistance phenotype negatively predicted STa presence (OR: 0.19; 95% CI: 0.03, 0.77), while presence of upstream CHO had an increased, though insignificant (OR: 3.68; 95% CI: 0.66, 69.2) association with STa virulence presence.

When assessing the effect of phenotypic resistance on specific virulence genes, we found significant associations of resistance with presence of the STa virulence gene (Figure 3). Like the analysis of the “at least one virulence gene” outcome, the presence of a resistance phenotype decreased the likelihood of STa presence (OR: 0.19; 95% CI: 0.028, 0.77). CHO presence showed a strongly positive but statistically insignificant association (OR: 3.68; 95% CI: 0.66, 69.2). No other virulence genes were found to associate with any measured predictors (Table S4).

**Figure 4:**
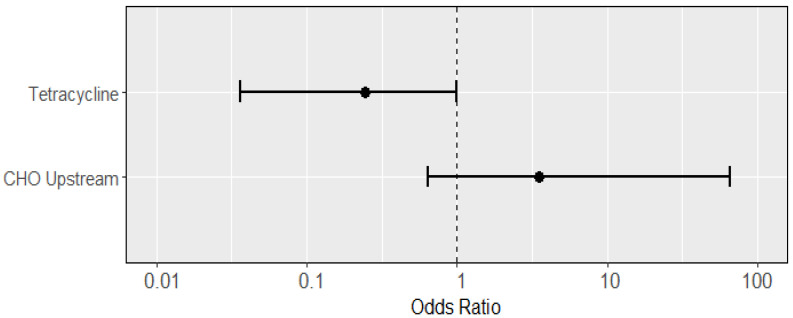
Odds ratios (dots; with 95% confidence intervals as lines) of predictors from a multiple logistic regression for an isolate having the STa virulence gene, using each type of resistance as a separate predictor (variables exhibiting complete or quasi-complete separation were dropped). Presence of tetracycline negatively predicted STa presence (OR: 0.24; 95% CI: 0.04, 0.99), while STa presence was not predicted by CHO presence (OR: 3.53; 95% CI: 0.64, 66.3), though it trended positively.

Analyzing individual phenotypes once more (Figure 4), tetracycline resistance was the only significantly associated variable, reducing the likelihood of STa presence (OR: 0.24; 95% CI: 0.036, 0.99). Resistance to amoxicillin-clavulanate, ampicillin, cefoxitin or ceftriaxone, chloramphenicol, ciprofloxacin or levofloxacin, sulfamethoxazole-trimethoprim, multi-drug resistance, and ESBL production displayed complete or quasi-complete separation, and were thus dropped. Again, CHO presence was strongly associated, but statistically insignificant (OR: 3.53; 95% CI: 0.64, 66.3).

## 4. Discussion

This work found a negative association between AMR and virulence in surface-water *E. coli*. The relationship seen in the general model might be largely explained by the negative relationship seen between tetracycline resistance and STa, as no other individual phenotypes or genes showed significant associations. However, it should be noted that tetracycline resistance was by far the most prevalent resistance phenotype evaluated (44.3%, with the next highest being ampicillin at 12.6%). It is thus possible that negative associations with tetracycline resistance are true for resistance more broadly, but that only tetracycline resistance was prevalent enough to reach statistical significance. This is less likely as an explanation for the STa associations, as STa was not a particularly prevalent virulence gene. Furthermore, we did not find significant evidence of a relationship between CHO presence and virulence in this dataset. Yet, the large positive effect size may suggest an underlying trend that fell shy of statistical significance, especially because the lower prevalence of resistance observed at background sites limited the background sample size (*n* = 34; 17 pairs), thus limiting statistical power [21]. This would align with previous work that pointed to connections between animal agriculture and the environmental dissemination of pathogens [33,34].

There is no standard panel of genes used to assess the prevalence of virulence in surface-water *E. coli*, and results thus vary in the literature, ranging from 10% to 87.5% of isolates carrying at least one virulence gene [15,35]. Our panel was selected to include genes from various *E. coli* serotypes, important to both human and swine infections and present on plasmids and/or chromosomes. We selected genes of interest in an area impacted by animal agriculture, resulting in a panel similar to that used in a study of the Minjiang River, which is also impacted by animal agriculture [20]. Though the authors tested twelve instead of seven virulence genes, their panel shared the *eae* gene and the toxin genes *ast*A and *est*A with our panel and included other toxin genes and various fimbriae (similar to our *fim*H). In the Minjiang River study, at least one virulence gene was found in 24% of studied isolates, remarkably close to our 24.7%, though it may be expected to find more virulence when using a larger panel. The authors found *ast*A in 10.7% of isolates (13.8% in our study) and *est*A in 3.7% of isolates (6.3% here). Outside of *fim*H, *ast*A and *est*A were the two most prevalent virulence genes in both studies.

The fimbrial adhesin gene *fim*H has previously been detected in surface-water *E. coli*, but the high level of *fim*H seen in this study appears to be uncommon [36]. It is unclear what caused this dominance over other virulence genes. Our confirmation of PCR product length via gel electrophoresis makes lack of primer specificity an unlikely culprit. One hypothesis is the potential connection between animal agriculture and urinary tract infections (UTIs). *fim*H in *E. coli* is associated with UTIs and recent research has associated animal food products with UTIs via widespread *E. coli* sequence types like ST131 [37,38,39,40]. It may therefore be possible for uropathogenic *E. coli* or their genetic elements to have originated from farm animals in our study area and then traveled to surface waters. Interestingly, fimbrial genes in the Minjiang River study belonged to fimbrial systems not associated with urinary infections and were detected in only a handful of isolates. Of course, the current study found *fim*H at an equally high prevalence in both CHO-associated and background sites, challenging this hypothesis. Nevertheless, further investigation of *fim*H prevalence in surface-water *E. coli* is warranted, as is investigation of the potential connection between commercial agriculture and uropathogenic *E. coli*.

This project’s strengths in study design and data quality control distinguish it from others of its kind. Primarily, we were able to control for some of the variables that commonly limit the generalizability of environmental microbiological work. A matching approach allowed us to account for spatial and temporal variables, including seasonality and rainfall [41,42]. Another common concern with generalizing from environmental samples is the potential presence of genetic clones. Though selecting separate colonies during the isolation process helps prevent this, it does not necessarily confirm that isolates are genetically distinct and thus representative of the full breadth of strain diversity. Our method of collapsing potential clones into single representative pairs moves a step further in reducing clonality in the dataset. Results from the collapsed (presented here and in Table S4) and uncollapsed (Table S1) datasets generally produced similar results. One exception is that a negative association appeared in the uncollapsed dataset between EAST-1 and CHO presence. However, since the size of the background (i.e., no upstream CHO) group was fairly small (*n* = 42; 21 pairs) due to a lower prevalence of observed resistance, one group of isolates from the same sample possessing the same virulence and AMR profiles (i.e., assumed clones) made up 50% of the EAST-1 background positives. In small datasets with low-frequency outcomes, a collapsing method limits bias resulting from such amplification of positives. Finally, many studies of AMR and virulence genes from environmental samples exclusively use molecular methods, making it difficult to tell what bacteria are carrying the genes and whether the bacteria are viable [43]. One advantage of this study is that all tested bacteria were originally isolated by culture. The results reported in this paper are all associated with viable bacteria.

Our study does not provide direct evidence for a mechanism behind the negative AMR–virulence relationship, but exploring the topic is nonetheless valuable. General negative associations between AMR and virulence might be explained by fitness costs. While discussions of fitness cost typically focus on costs of AMR, we postulate that the biological cost of plasmids may make possession of separate AMR and virulence plasmids unfavorable [14,44]. This would be particularly relevant in environmental contexts, where virulence genes are unlikely to benefit survival and conditions are typically more stressful than in nutrient-rich contexts (e.g., biological tissues and wastewater treatment plants). Recent research indicates that nutrient levels control the fitness cost of plasmids in *Enterococcus faecium* in surface water [45]. It is thus plausible that fitness costs exhibited by simultaneous possession of virulence and AMR would not emerge until a pathogen passed from its host into a secondary habitat. Additionally, some researchers have suggested plasmid incompatibility as an explanation for negative associations seen between AMR and the concurrent presence of STa and a heat-labile toxin, LT [46,47]. In the absence of sufficient selective pressures, plasmids with identical origins of replication often prevent one another from persisting in a bacterial community. However, little is known about which plasmids predominate in surface-water *E. coli*, so the likelihood of this explanation is unclear.

Associations between tetracycline resistance and STa in the literature differ for different tetracycline resistance genes. Research on *E. coli* from swine found a positive association between STa (*est*A) and *tet*A, but a negative association between STa and *tet*B [48]. Furthermore, a plasmid, pTC, has been identified in swine *E. coli* that carries STa, *tet*R, *tet*A, and *tet*C [34]. While this would support a positive association between these genes, this is a complex plasmid with many insertion sequences and transposases surrounding the virulence and resistance genes. It may thus be conceivable for these genes to separate or be present in competing genetic elements. More recent work on AMR and virulence plasmids in pigs found ARGs and virulence genes to generally exist on different plasmids [49]. It is also uncertain if the relationships and mechanisms seen in swine hold true in the environment. Dozens of different tetracycline resistance genes exist, and many of these genes have been detected in environmental waters and agricultural contexts, so it is difficult to predict which ones would be most likely in our study area [50,51]. Genetic approaches like PCR or sequencing on the isolates would be useful to identify the subtype and genetic context of the tetracycline resistance, allowing for a better understanding of associations with other genes.

This study possesses a few notable limitations. First, the presence of virulence genes does not confirm functional virulence. We cannot infer if the studied isolates represent immediate threats to human or animal health. That said, these genes indicate the relative virulence potential of the resistant and susceptible groups and are therefore useful for comparing the two. Relying on genotypic approaches remains popular in the field of environmental AMR for this reason, though, and can play a valuable role in environmental AMR monitoring [52]. In contrast, while phenotypic AMR data represent the actual resistant ability of the isolates, they do not reveal which genes are present. Since the AMR–virulence relationship depends on genetic mechanisms, this information gap prevents us from investigating the mechanisms underlying the observed relationships. Second, due to a lack of mechanistic evidence provided by this research, we cannot comment on delayed risks that may be associated with environmental AMR. We recognize that AMR dissemination is complex and does not require co-occurrence with virulence in the environment to pose a risk. Indeed, ample evidence exists for the exchange of AMR genes between environmental and clinical bacteria [53]. Finally, it is generally understood that virulence genes, resistances, and the mechanisms that associate them all vary by species [54]. It is therefore difficult to extend the results of this work to other species. Still, *E. coli* appears a suitable organism for developing our understanding of AMR and virulence in the environment, as it is a common model organism used as the basis for many environmental regulations.

More work is needed to develop a detailed understanding of the AMR–virulence relationship in the environment. Firstly, achieving enough statistical power to detect differences among infrequent resistances and virulence genes necessitates studies with larger sample sizes. Secondly, identifying individual mechanisms behind the observed associations will be critical, and may be pursued by a fully genetic approach. For instance, determining which tetracycline resistance gene is most common among our isolates may help clarify the STa–*tet* relationship. An abundance of the *tet*B genotype might be expected given the previous negative association with STa. One interesting route of study would involve assessing the presence of the pTC plasmid, or related mobile genetic elements, in these isolates. Given the association of this plasmid with swine-related *E. coli*, this may have interesting implications for microbial source tracking.

## 5. Conclusions

This work revealed a negative association between antimicrobial resistance and virulence in *E. coli* from surface waters in eastern North Carolina, an area of dense food animal production. We also found a negative association between tetracycline resistance and the heat-stable enterotoxin STa, though it is not clear if this was the cause of the general association. These findings add to the scant body of knowledge on the AMR–virulence relationship in water, helping us understand what happens when resistant organisms enter aquatic systems. With or without the co-occurrence of virulence genes, the dissemination of antibiotic-resistant bacteria and genes in environmental systems is an area of public health concern. Improvements to fecal waste management in CHOs may be helpful in reducing the introduction and spread of resistance elements. This work also reflects the value of pursuing ecological questions to understand the dissemination of AMR. We believe that perspectives from the field of microbial ecology will be important to inform ongoing health discussions inherent to AMR.

## Figures and Tables

**Table 1:** PCR conditions used for virulence genes, modified from references. “#” means “number”.

Target	Initial Denaturing	Denaturing	Annealing	Extension	# of Cycles	Final Extension	Reference
*bfp*	94 °C, 3 min	94 °C, 30 s	60 °C, 30 s	72 °C, 30 s	30	72 °C, 5 min	[24]
*fim*H	98 °C, 30 s	98 °C, 30 s	52 °C, 30 s	72 °C, 30 s	30	72 °C, 5 min	[25]
*cnf*-1	94 °C, 2 min	94 °C, 30 s	45 °C, 30 s	72 °C, 40 s	38	72 °C, 5 min	[26]
STa (*est*A)	94 °C, 2 min	94 °C, 30 s	48 °C, 30 s	72 °C, 20 s	35	72 °C, 5 min	[26]
EAST-1 (*ast*A)	95 °C, 2 min	95 °C, 30 s	55 °C, 30 s	72 °C, 30 s	30	72 °C, 5 min	[27]
*eae*	94 °C, 5 min	94 °C, 30 s	65 °C, 30 s	68 °C, 75 s	40	68 °C, 7 min	[28]
*hly*A	94 °C, 5 min	94 °C, 30 s	65 °C, 30 s	68 °C, 75 s	40	68 °C, 7 min	[28]

**Table 2:** Primer sequences and product sizes for virulence genes.

Target		Primer Sequence (5′-3′)	Size (bp)	Reference
*bfp*	Forward Reverse	AATGGTGCTTGCGCTTGCTGC GCCGCTTTATCCAACCTGGTA	326	[24]
*fim*H	Forward Reverse	GATCTTTCGACGCAAATC CGAGCAGAAACATCGCAG	389	[29]
*cnf*-1	Forward Reverse	GAACTTATTAAGGATAGT CATTATTTATAACGCTG	543	[30]
STa (*est*A)	Forward Reverse	TCCGTGAAACAACATGACGG ATAACATCCAGCACAGGCAG	244	[31]
EAST-1 (*ast*A)	Forward Reverse	CCATCAACACAGTATATCCGA GGTCGCGAGTGACGGCTTTGT	111	[32]
*eae*	Forward Reverse	CATTATGGAACGGCAGAGGT ACGGATATCGAAGCCATTTG	375	[28]
*hly*A	Forward Reverse	GCGAGCTAAGCAGCTTGAAT CTGGAGGCTGCACTAACTCC	199	[28]

**Table 3:** Virulence gene prevalence of isolated *E. coli*.

	N	*bfp*+	*fim*H+	*cnf*-1+	STa+	EAST-1+	*eae*+	*hly*A+	At Least 1+ (Excluding *fim*H)
CHO sites									
Resistant	70	1 (1.4%)	63 (90.0%)	2 (2.9%)	2 (2.9%)	7 (10.0%)	0 (0.0%)	2 (2.9%)	13 (18.6%)
Susceptible	64	0 (0.0%)	61 (95.3%)	3 (4.7%)	8 (12.5%)	10 (15.6%)	1 (1.6%)	0 (0.0%)	20 (31.3%)
Total	134	1 (0.7%)	124 (92.5%)	5 (3.7%)	10 (7.5%)	17 (12.7%)	1 (0.7%)	2 (1.5%)	33 (24.6%)
Background sites									
Resistant	17	0 (0.0%)	17 (100.0%)	1 (5.9%)	0 (0.0%)	1 (5.9%)	0 (0.0%)	0 (0.0%)	2 (11.8%)
Susceptible	23	0 (0.0%)	21 (91.3%)	2 (8.7%)	1 (4.3%)	6 (26.1%)	1 (4.3%)	0 (0.0%)	8 (34.8%)
Total	40	0 (0.0%)	38 (95.0%)	3 (7.5%)	1 (2.5%)	7 (17.5%)	1 (2.5%)	0 (0.0%)	10 (25.0%)
Total (CHO + Background)									
Resistant	87	1 (1.1%)	80 (92.0%)	3 (3.4%)	2 (2.3%)	8 (9.2%)	0 (0.0%)	2 (2.3%)	15 (17.2%)
Susceptible	87	0 (0.0%)	82 (94.3%)	5 (5.7%)	9 (10.3%)	16 (18.4%)	2 (2.3%)	0 (0.0%)	28 (32.2%)
Total	174	1 (0.6%)	162 (93.1%)	8 (4.6%)	11 (6.3%)	24 (13.8%)	2 (1.1%)	2 (1.1%)	43 (24.7%)

## Data Availability

Raw and processed data and R code used for data analyses are available in the Mendeley Data repository at https://doi.org/10.17632/mrwrszgg4y.1 [55].

## Supplementary Materials

The following supporting information can be downloaded at: https://www.mdpi.com/article/10.3390/microorganisms11112647/s1, Figure S1: Examples of plates from our AMR testing via Kirby–Bauer disk diffusion; Table S1: Beta coefficients and 95% confidence intervals for regressions on the uncollapsed dataset; Table S2: Test for multicollinearity; Table S3: Key assigning numbers to each group of models; Table S4: Beta coefficients and 95% confidence intervals for regressions on the collapsed dataset; Table S5: Beta coefficients and 95% confidence intervals for regressions on the collapsed dataset that used “at least 1 virulence” as the outcome, now including *fim*H.
