# Supplementary material for: Relating Antimicrobial Resistance and Virulence in Surface-Water E. coli
Source: Microorganisms. 2023 Oct 28;11(11):2647. doi: 10.3390/microorganisms11112647 (PMC10673096; doi:10.3390/microorganisms11112647)
Supplement: Supplementary file 1 [file microorganisms-11-02647-s001.zip › microorganisms-2666576-supplementary.pdf]

SUPPLEMENTAL INFORMATION

Title: Relating Antimicrobial Resistance and Virulence in Surface Water *E. coli*

Authors: Connor D. LaMontagne, Elizabeth C. Christenson-Diver, Anna T. Rogers, Megan E. Jacob, Jill R. Stewart

2023

For publication in *Microorganisms*

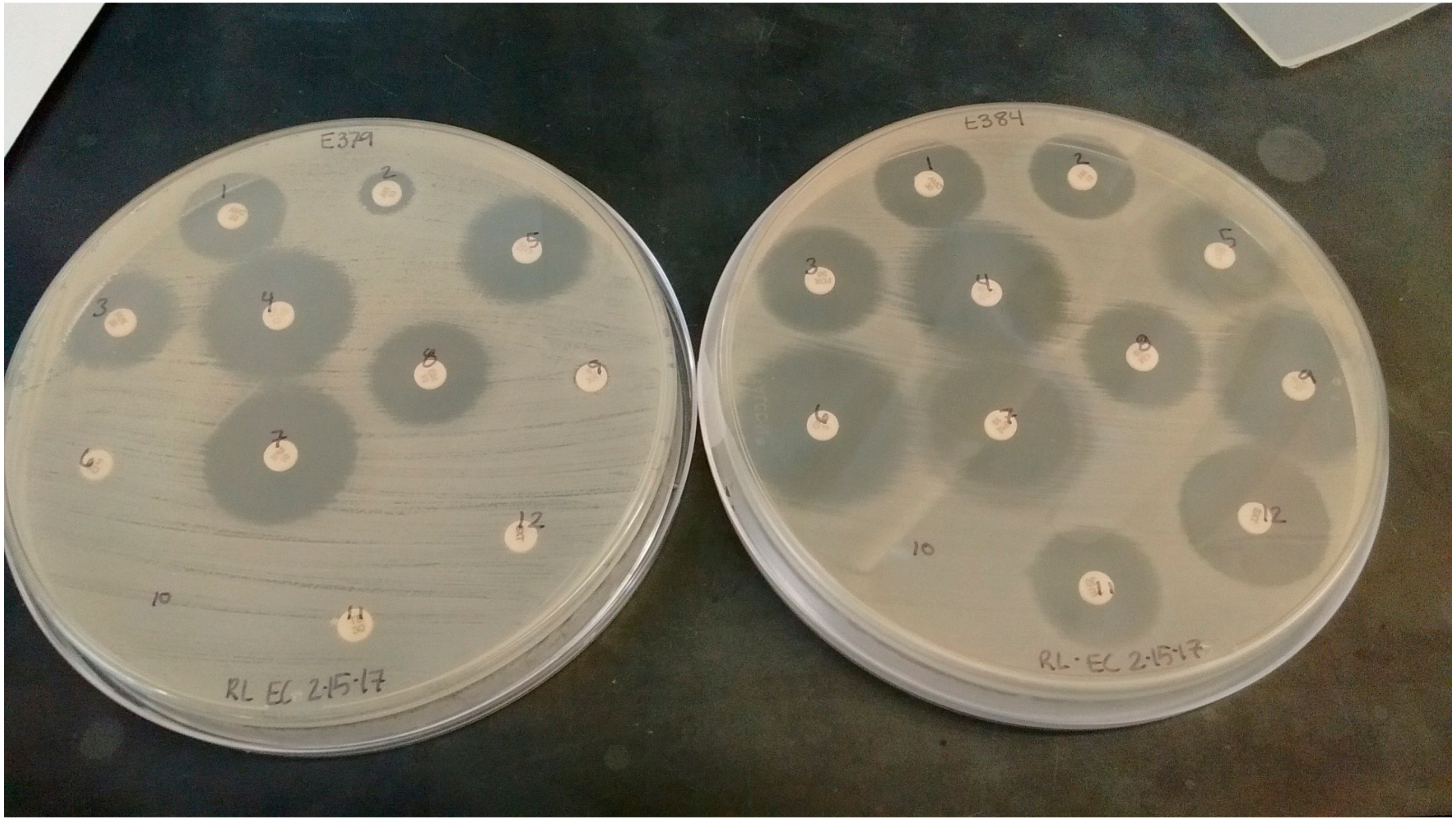

Figure S1. Examples of plates from our AMR testing via Kirby-Bauer disc diffusion. Resistance was assessed via the size of the clearing zone produced around each antibiotic disc, as determined by 2017 CLSI guidelines.

Table S1. Beta coefficients and 95% confidence intervals for regressions on the uncollapsed dataset. Models with the "at least 1 virulence" outcome do not include *fim*H here. Empty boxes indicate predictors without enough positive outcomes to model. Results from model group 1 are highlighted orange, model 2 yellow, model 3 blue, and model 4 grey. ** denotes $p < 0.05$.

		"At least 1 resistance" (model 1 and model 3)		"Individual resistances" (model 2 and model 4)											
		At least 1 resistance	CHO presence	Amoxicillin-clavulanate	Ampicillin	Cefoxitin or Ceftriaxone	Chloramphenicol	Imipenem	Ciprofloxacin or Levofloxacin	Gentamicin	Tetracycline	Sulfamethoxazole -Trimethoprim	Extended-Spectrum Beta Lactamase	Multi-drug	CHO presence
"At least 1 virulence" (model 1 and model 2)	At least 1 virulence	-0.39 (-0.99, 0.19)	-0.39 (-1.05, 0.29)	0.15 (-2.89, 2.27)	-0.36 (-1.53, 0.63)	-	-	-	-	-	-0.40 (-1.04, 0.20)	1.44 (-0.30, 3.12)	-	-	-0.42 (-1.09, 0.26)
"Individual virulences" (model 3 and model 4)	*bfp*	-	-	-	-	-	-	-	-	-	-	-	-	-	-
	*fim*H	-0.57 (-1.78, 0.52)	-0.46 (-2.35, 0.89)	-1.20 (-3.48, 1.92)	-0.51 (-1.88, 1.21)	-	-	-	-	-	0.24 (-0.87, 1.41)	-	-	-	-0.53 (-2.42, 0.82)
	cnf-1	-0.58 (-2.17, 0.79)	-1.00 (-2.37, 0.43)	-	-	-	-	-	-	-	-0.39 (-1.98, 0.98)	-	-	-	-1.03 (-2.41, 0.40)
	STa	-1.90** (-3.78, -0.55)	1.52 (-0.15, 4.44)	-	-	-	-	-	-	-	-1.68** (-3.57, -0.33)	-	-	-	1.46 (-0.20, 4.38)
	EAST-1	-0.23 (-0.97, 0.50)	-0.84** (-1.60, -0.057)	-	-0.77 (-2.63, 0.51)	-	-	-	-	-	-0.10 (-0.86, 0.63)	-	-	-	-0.81** (-1.58, -0.027)
	eae	-	-0.98 (-4.23, 2.26)	-	-	-	-	-	-	-	-	-	-	-	-1.27 (-4.51, 1.96)
	*hly*A	-	-	2.05 (-1.30, 5.10)	2.12 (-0.88, 5.38)	-	-	-	-	-	0.35 (-2.21, 3.51)	-	-	-	-

Table S2. Test for multicollinearity among predictor variables via Pearson's r. r > 0.5 (highlighted yellow) represents a moderate correlation and r > 0.8 (highlighted red) represents a severe correlation. No substantial correlations were found between relevant predictors.

	CHO Upstream	At Least 1 Resistance	Amoxicillin-clavulanate	Ampicillin	Cefoxitin or Ceftriaxone	Chloramphenicol	Imipenem	Ciprofloxacin or Levofloxacin	Gentamicin	Tetracycline	Sulfamethoxazole-Trimethoprim	ESBL	Multi-drug
CHO Upstream	1	0.08	-0.01	0.08	0.04	0.09	NA	0.06	NA	0.07	0.09	0.1	0.11
At Least 1 Resistance	0.08	1	0.15	0.38	0.2	0.17	NA	0.11	NA	0.89	0.17	0.28	0.3
Amoxicillin-clavulanate	-0.01	0.15	1	0.29	0.75	-0.03	NA	-0.02	NA	0.02	-0.03	0.54	0.52
Ampicillin	0.08	0.38	0.29	1	0.45	0.35	NA	0.28	NA	0.11	0.25	0.68	0.65
Cefoxitin or Ceftriaxone	0.04	0.2	0.75	0.45	1	-0.04	NA	-0.02	NA	0.05	-0.04	0.72	0.69
Chloramphenicol	0.09	0.17	-0.03	0.35	-0.04	1	NA	0.3	NA	0.19	0.59	0.47	0.58
Imipenem	NA	NA	NA	NA	NA	NA	1	NA	NA	NA	NA	NA	NA
Ciprofloxacin or Levofloxacin	0.06	0.11	-0.02	0.28	-0.02	0.3	NA	1	NA	0.12	0.3	0.38	0.36
Gentamicin	NA	NA	NA	NA	NA	NA	NA	NA	1	NA	NA	NA	NA
Tetracycline	0.07	0.89	0.02	0.11	0.05	0.19	NA	0.12	NA	1	0.19	0.19	0.2
Sulfamethoxazole-Trimethoprim	0.09	0.17	-0.03	0.25	-0.04	0.59	NA	0.3	NA	0.19	1	0.34	0.46
ESBL	0.1	0.28	0.54	0.68	0.72	0.47	NA	0.38	NA	0.19	0.34	1	0.96
Multi-drug	0.11	0.3	0.52	0.65	0.69	0.58	NA	0.36	NA	0.2	0.46	0.96	1

Table S3. Key assigning numbers to each group of models. Models with the "at least 1 virulence" outcome do not include *fim*H here.

Model Group	Outcome Variable	Predictor Variables (after removing variables exhibiting separation)
1	At least 1 virulence	Any resistance, CHO presence
2	At least 1 virulence	Ampicillin, amoxicillin-clavulanate, tetracycline, CHO presence
3	*bfp*	(No usable variable)
	*fim*H	Any resistance, CHO presence
	cnf-1	Any resistance, CHO presence
	STa	Any resistance, CHO presence
	EAST-1	Any resistance, CHO presence
	eae	CHO presence
	*hly*A	(No usable variable)
4	*bfp*	(No usable variable)
	*fim*H	Ampicillin, amoxicillin-clavulanate, tetracycline, CHO presence
	cnf-1	Tetracycline, CHO presence
	STa	Tetracycline, CHO presence
	EAST-1	Ampicillin, tetracycline, CHO presence
	eae	CHO presence
	*hly*A	Ampicillin, tetracycline

Table S4. Beta coefficients and 95% confidence intervals for regressions on the collapsed dataset. Models with the "at least 1 virulence" outcome do not include *fim*H here. Empty boxes indicate predictors without enough positive outcomes to model. Results from model group 1 are highlighted orange, model 2 yellow, model 3 blue, and model 4 grey. ** denotes $p < 0.05$

		"At least 1 resistance" (model 1 and model 3)		"Individual resistances" (model 2 and model 4)											
		At least 1 resistance	CHO presence	Amoxicillin-clavulanate	Ampicillin	Cefoxitin or Ceftriaxone	Chloramphenicol	Imipenem	Ciprofloxacin or Levofloxacin	Gentamicin	Tetracycline	Sulfamethoxazole -Trimethoprim	Extended-Spectrum Beta Lactamase	Multi-drug	CHO presence
"At least 1 virulence" (model 1 and model 2)	At least 1 virulence	-0.82** (-1.56, -0.12)	0.059 (-0.74, 0.92)	0.27 (-2.83, 2.58)	-0.36 (-1.72, 0.76)	-	-	-	-	-	-0.77** (-1.53, -0.047)	-	-	-	0.061 (-0.74, 0.92)
"Individual virulences" (model 3 and model 4)	*bfp*	-	-	-	-	-	-	-	-	-	-	-	-	-	-
	*fim*H	-0.33 (-1.59, 0.84)	-0.39 (-2.29, 1.00)	-1.54 (-4.09, 1.64)	-0.10 (-1.69, 2.07)	-	-	-	-	-	0.54 (-0.67, 1.92)	-	-	-	-0.46 (-2.36, 0.93)
	cnf-1	-0.48 (-2.09, 0.96)	-0.69 (-2.15, 0.93)	-	-	-	-	-	-	-	-0.24 (-1.85, 1.20)	-	-	-	-0.71 (-2.17, 0.90)
	STa	-1.66** (-3.57, -0.26)	1.30 (-0.41, 4.23)	-	-	-	-	-	-	-	-1.40** (-3.31, -0.0096)	-	-	-	1.26 (-0.45, 4.19)
	EAST-1	-0.77 (-1.73, 0.10)	-0.31 (-1.25, 0.71)	-	-0.38 (-2.27, 0.97)	-	-	-	-	-	-0.71 (-1.71, 0.19)	-	-	-	-0.30 (-1.24, 0.72)
	eae	-	-1.22 (-4.47, 2.01)	-	-	-	-	-	-	-	-	-	-	-	-1.22 (-4.47, 2.01)
	*hly*A	-	-	-	1.97 (-1.30, 5.26)	-	-	-	-	-	-0.025 (-3.31, 3.25)	-	-	-	-

Table S5. Beta coefficients and 95% confidence intervals for regressions on the collapsed dataset that used "at least 1 virulence" as the outcome, now including *fim*H.

		"At least 1 resistance" (model 1)		"Individual resistances" (model 2)											
		At least 1 resistance	CHO presence	Amoxicillin-clavulanate	Ampicillin	Cefoxitin or Ceftriaxone	Chloramphenicol	Imipenem	Ciprofloxacin or Levofloxacin	Gentamicin	Tetracycline	Sulfamethoxazole -Trimethoprim	Extended-Spectrum Beta Lactamase	Multi-drug	CHO presence
**"At least 1 virulence" (model 1 and model 2)**	At least 1 virulence	-0.54 (-1.92, 0.70)	-1.10 (-4.02, 0.61)	-1.63 (-4.21, 1.57)	-0.16 (-1.77, 2.03)	-	-	-	-	-	0.42 (-0.85, 1.82)	-	-	-	-1.18 (-4.11, 0.53)
